# Therapeutic Use of an Inhaled Drug Delivery in Pulmonary Hypertension: A Review

**DOI:** 10.7759/cureus.30134

**Published:** 2022-10-10

**Authors:** Unnati Kumbhare, Pallavi Yelne, Sanket Tekale

**Affiliations:** 1 Department of Internal Medicine, Jawaharlal Nehru Medical College, Datta Meghe Institute of Medical Sciences (Deemed University), Wardha, IND; 2 Department of Medicine, Jawaharlal Nehru Medical College, Datta Meghe Institute of Medical Sciences (Deemed University), Wardha, IND

**Keywords:** targeted drug delivery, prostacyclin, newer drugs, inhalation route, pulmonary artery hypertension

## Abstract

Pulmonary arterial hypertension (PAH) is a serious condition in which there is increased blood pressure in arteries of the lungs (pulmonary arteries). The therapies or drugs for PAH have expanded with the revelation of three key pathological processes - encompassing prostacyclin, nitric oxide (NO), and endothelin pathways. An outlook for patients suffering from PAH is still mediocre amidst recent advancements. The evolution of pre-clinical and clinical research on PAH has facilitated the identification of several new targeted therapies for the disease. In this article, we examine recent data on new pulmonary hypertension physiological pathways, primarily concentrating on administering drugs through the inhalation route and their effects. Although they have been given clinical use approval, medications based on these routes are presently being studied in clinical or pre-clinical settings. To confirm these innovative medicines' therapeutic efficacy and safety, extensive clinical trials are needed.

## Introduction and background

Pulmonary artery hypertension (PAH) is caused due to cardiopulmonary arterial blood pressure disturbance that sets off a chain of events at the junction of capillaries and alveoli. Pulmonary hypertension (PH) can be classified as pre- or post-capillary PH. Precapillary PH occurs due to a primary elevation of pressure in the pulmonary precapillary PH (e.g., PAH), while post-capillary PH occurs due to elevations of pressure in the pulmonary venous and pulmonary capillary systems (pulmonary venous hypertension) [1]. There may be a rise of over 25 mmHg pressure in pulmonary artery. The higher flow resistance brings on diastolic dysfunction in the PAH and the overload that results in the right ventricle which induces fibrosis, hypertrophy, and hyperplasia in the cardiopulmonary vasculature. All of the occurrences above result in right-sided heart failure, a substantial cause of death in PAH individuals. The mean pulmonary artery pressure (mPAP) is elevated by around 30 mmHg hemodynamically, while usual left ventricular refilling pressures are maintained. The mPAP, however, is 25 mmHg at rest [1]. Elevated pulmonary vascular resistance (PVR), brought on due to unchecked pulmonary vascular restructuring, is a pathologic characteristic of this condition. High PVR causes right ventricular hypertrophy (RVH), persistent vasoconstriction, rise in gradual pulmonary arterial pressure (PAP), and eventually cardiac failure and mortality. Some mechanisms for enhanced PVR in PAH include vasoconstriction, remodeling, and thrombosis [2]. Other cell types found inside the walls of the pulmonary arteries include pulmonary artery endothelial cells (PAECs), pulmonary artery smooth muscle cells (PASMCs), fibroblast, inflammatory cells, and platelets are all thought to play a role in the diseased state. PAECs make up the intimal layer of the artery, PASMCs make up the medial layer, and fibroblasts make up most of the adventitial layer. The genesis and spread of PAH have been associated with the dysfunction of each of the following cell types. The tone of the pulmonary arteries alters when damaged by shear strength, prolonged constriction, hypoxic state, inflammatory conditions, or toxins because they generate fewer vasodilators, such as prostacyclin and nitric oxide (NO) and vasoconstrictors, including endothelin-1 (ET-1) [2]. The inequity of endothelium-derived vasoconstrictors and vasodilators, coupled with the suppression of voltage-gated k+ channels in PASMCs, results in abnormal PASMC contractility. This, in turn, opens voltage-gated calcium channels and raises the saturation of intracellular calcium. Defective cells may also be unable to produce molecules that normally limit PASMC proliferation, such as apelin, or they may release compounds that promote PASMC proliferation, such as fibroblast growth factor 2 (FGF-2). Unchecked PASMC proliferation, contraction, apoptosis resistance, and neointimal development result in vasculature restructuring and obstructing lesions that reduce the lumen area of various arteries and restrict blood circulation. Prostacyclin and their analogs, the prostacyclin route, soluble guanylate cyclase modulators, the endothelin pathway, phosphodiesterase-5 inhibitors (PDE5), and inhaled NO are some of the main mechanisms that are targeted by PAH therapeutics [3]. Endothelial progenitor cells, the RhoA/Rho kinase, the vasoactive intestinal peptide (VIP), and micro-RNA (ribonucleic acid) are new molecular pathways that target PAH treatments. Due to its better delivery abilities, particularly the ability to transport specific, relatively lower dosage molecules to deeper parts of the lungs, drugs targeting the pulmonary alveoli in the lungs have served as the primary method. It also has a benefit when systemic adverse effects must be minimized, and a quicker beginning of action is necessary. Targeting such small targets requires a thin pulmonary epithelium because it has a greater surface area. However, equally crucial is the efficient dispersal of powders to create an aerosol mist that is safe for inhaling [3]. These molecules must withstand the pre-formulation stresses by spray drying, grinding, or freeze drying. The surface features of the powder formulation are also very important since they affect how many medications are accessible to the junction of the arterial and pulmonary arteries.

## Review

Classification of pulmonary hypertension 

**Figure 1:**
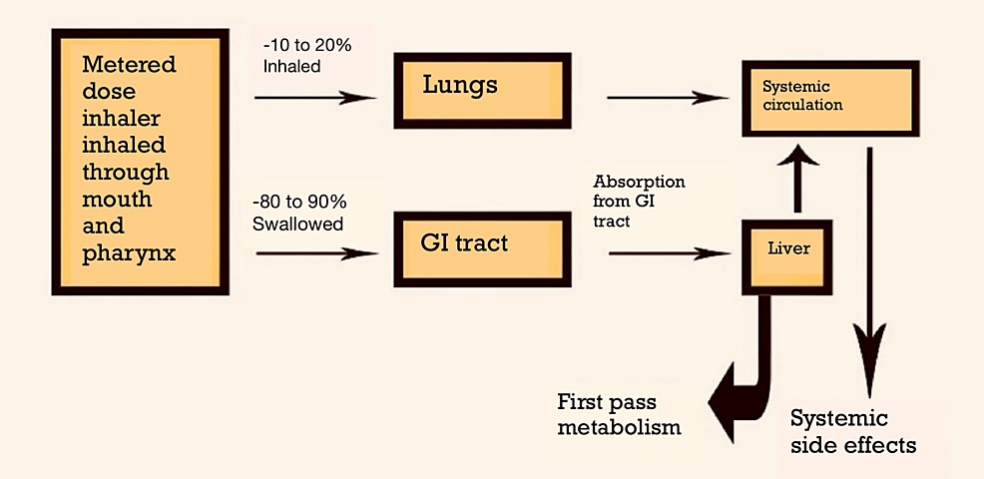
Diagram showing drug distribution through inhalational route. GI: gastrointestinal tract

Pulmonary arterial hypertension (PAH) is categorized as primary and secondary PAH depending on whether certain principles or risks are present or absent. Primary PAH is hypertension that has no underlying causes. The main objective of the clinical categorization of PAH that experts have suggested is to classify the contributing factors of PAH based on similar pathophysiology and clinical manifestations [4]. Symptoms include dyspnea on exertion, fatigue, syncope, angina chest pain, and hemoptysis and present with signs of raised jugular venous pressure, pedal edema, holosystolic murmur, wide split of S2, and hepatomegaly. Since the pathophysiological changes have already started by the time patients are evaluated because the early symptoms of PAH are not always obvious, and more people are discovered in the intermediate stages. According to a study by Sarah et al., pulmonary arterial hypertension is a family of pulmonary vascular illnesses, which can result in heart failure or possibly cause mortality [5]. Thus, administration of drugs through inhalation route may be helpful in achieving the desired results in therapy of PAH. Figure 1 shows the distribution of drugs through inhalation route [6].

Therapeutic targets for the management of PAH

Prostacyclin Pathway

Arachidonic acid is converted into prostaglandin I_2_ (PGI_2_, prostacyclin) by the enzyme prostacyclin synthase in an arterial endothelial layer. PGI_2_ causes relaxation of pulmonary artery smooth muscle cells (PASMCs) by increasing quantity of cyclic adenosine monophosphate (cAMP) and inhibiting growth of the medial layer of pulmonary arteries. A study showed that PGI_2_ synthase activity and its metabolites are diminished in the pulmonary circulation of people with PAH. As a result, individuals with PAH have received an exogenous injection of prostacyclin analogs [7]. Epoprostenol, the first prostacyclin - epoprostenol (Flolan®), was licensed by Food and Drug Administration (FDA) in 1995 for the management of PAH. It is administered by a central vein catheter connected to an intravenous (IV) infusion pump utilizing continuous IV infusion, which calls for careful planning and strict cleaning standards. According to the primary randomized clinical research, epoprostenol enhanced exercise performance, blood oxygenation, in PAH individuals having grades New York Heart Association (NYHA) class III and IV. Flushing, headaches, jaw discomfort, and diarrhea are frequent side effects. More serious issues might arise, mostly because of the delivery system. Catheter-related infections are the most serious mechanical complications, along with pump failure and catheter dislodgements. Hemodynamic measures often remained significantly impaired in many patients receiving long-term PGI_2_ treatment despite great clinical improvement due to infusion of epoprostenol [8]. A physiologically inert prostacyclin derivative with an approximate half-life of 30 min is iloprost (Ventavis®). The Aerosolized Iloprost Randomized (AIR) study found that after 12 weeks, individuals with PAH on NYHA class III or IV who used inhaled iloprost had improved their way of life, dyspnea assessments, hemodynamic parameters, and exercise tolerance. Inhaled medicine delivery has a less negative impact on systemic symptoms than IV medication administration. Regrettably, only a tiny portion of patients could be stabilized with this therapy during a follow-up [9]. Treprostinil (Tyvaso®), a more stable prostacyclin analog, can be given orally, intravenously, subcutaneously, or by inhalation. Sandifer et al. contrasted the effects of treprostinil delivered intravenously and by aerosol on sheep exposed to PAHs [10]. Although at a lowered dosage (250 ng/kg/min), aerosolized treprostinil was superior to IV treprostinil as a pulmonary vasodilator. Both mean pulmonary arterial pressure (mPAP) and peripheral vascular resistance (PVR) were reduced by treprostinil to baseline values. It has the longest half-life, three to four hours, compared to other prostacyclin analogs resulting in a longer dose interval when delivered through inhalation [10].

Nitric Oxide Pathway

Nitric oxide (NO) is an endogenous vasodilator and is produced when arginine is converted by NO synthase in pulmonary vascular endothelial cells. When NO enters the vascular smooth muscle, guanylate cyclase uses it to catalyze the formation of cyclic guanylate monophosphate (cGMP). Then, cGMP promotes the relaxation of smooth muscles in the artery wall and controls cellular proliferation and inflammation. In PAH, pulmonary endothelial cells lack NO synthase and produce less. NO alters the vascular tone and other cellular processes in the vessel wall [11]. Phosphodiesterase-5 (PDE5) is tadalafil, sildenafil, as well as soluble guanylate cyclase (sGC) stimulants, as it has long-term survival rates and can be combined with other agents for better outcomes, so they have been authorized for use in treating PAH since the 1990s (riociguat). By selectively inhibiting the PDE5 isoenzyme that catalyzes the breakdown of cGMP, phosphodiesterase-5 inhibitors (PDE5Is) are vasodilators that prolong and increase the effect of cGMP [12]. Riociguat works with endogenous NO by increasing sGC sensitivity to it by stabilizing the NO-sGC binding and directly inducing sGC to generate cGMP without regard to the presence of NO.

Endothelin Pathway

Endothelin (ET-1) is universally and primarily generated by pulmonary artery endothelial cells (PAECs), with minor contributions from PASMCs and fibroblasts [13]. Furthermore, several promoters, such as cytokines, hypoxic states, shear stresses, growth factors (GFs), and angiotensin II increases the synthesis of ET-1 after interacting with two different types of endothelin receptors (ET), including ET receptor A (ETA), which is primarily found on PASMCs, and ET receptor B (ETB), which is primarily found on vascular PAECs [14,15].

After interacting with two different types of endothelin receptors (ET), including ET receptor A (ETA), which is primarily found on PASMCs, and ET receptor B (ETB), which is primarily found on vascular PAECs and less on PASMCs [15]. Vascular smooth muscle cells (SMCs) become more active and increase when the ETA isoform is activated. Because the ETB receptors clear ET-1, this process may cause vasodilation to release prostacyclin and NO. All forms of human PAH were shown to have the ET route activated in all patient groups. Higher plasma and pulmonary arterial ET-1 expression levels were found in PAH patients, which strongly correlated with the disease's severity, including the degree of vascular remodeling [16]. Patients with PAH should take the dual endothelin receptor antagonist bosentan. Bosentan reaches maximal plasma concentrations following oral treatment after around three hours [17]. Ambrisentan, an orally selective ETA receptor antagonist and dual endothelin receptor agonist, is a drug that has a lower risk of liver toxicity [18]. Over two years, ambrisentan showed improved NYHA grading, life expectancy measures, and tolerance profiles in two significant, multicenter investigations known as ARIES-1 and 2 [19]. Table 1 gives an idea of the drug recommendations in PAH.

**Table 1:** Food and Drug Administration recommended drugs for treatment of pulmonary hypertension. NYHA: New York Heart Association; IV: intravenous; PO: by mouth

Drug	Class/indication	Method of administration	Dosage	Adverse effects
Epoprostenol, prostaglandin analog [8]	NYHA class III-IV	Intravenous (IV)	2 ng/kg/min continuous infusion, titrate to desired effect as tolerated	Hypotension, jaw pain, vomiting, diarrhea, headache, thrombocytopenia, bloodstream infection, flushing
Bosentan, endothelin receptor antagonist	NYHA class III-IV	PO (by mouth)	62.5 mg twice a day for four weeks, then 125 mg twice a day	Hypotension, flushing, vomiting, peripheral edema, headache, hepatotoxicity thrombocytopenia, teratogenesis
Treprostinil, prostacyclin analog [10]	NYHA class II-IV	Subcutaneous (SC), IV	1.25 ng/kg/min continuous infusion, titrate to desired effect as tolerated	Infusion site pain, site infection, bloodstream, infection, flushing, diarrhea, nausea, jaw pain, headache
Iloprost, prostacyclin analog [9]	NYHA class III-IV	Inhaled	2.5-5 μg, six to nine times a day	Cough, syncope, hypotension, flushing, headache, trismus
Sildenafil, phosphodiesterase-5 inhibitor [12]	NYHA class I-IV	PO	20 mg, three times a day	Flushing, diarrhea, indigestion, nasal congestion dizziness, headache
Ambrisentan, endothelin receptor antagonist [18]	NYHA class II-III	PO	5-10 mg once a day	Headache, peripheral edema, rhinosinusitis, anemia, teratogenesis
Treprostinil, prostacyclin analog	NYHA class III	Inhaled	18 μg, four times a day	Cough, headache, pharyngolaryngeal, pain, throat infection, syncope

Other Pathways

**Figure 2:**
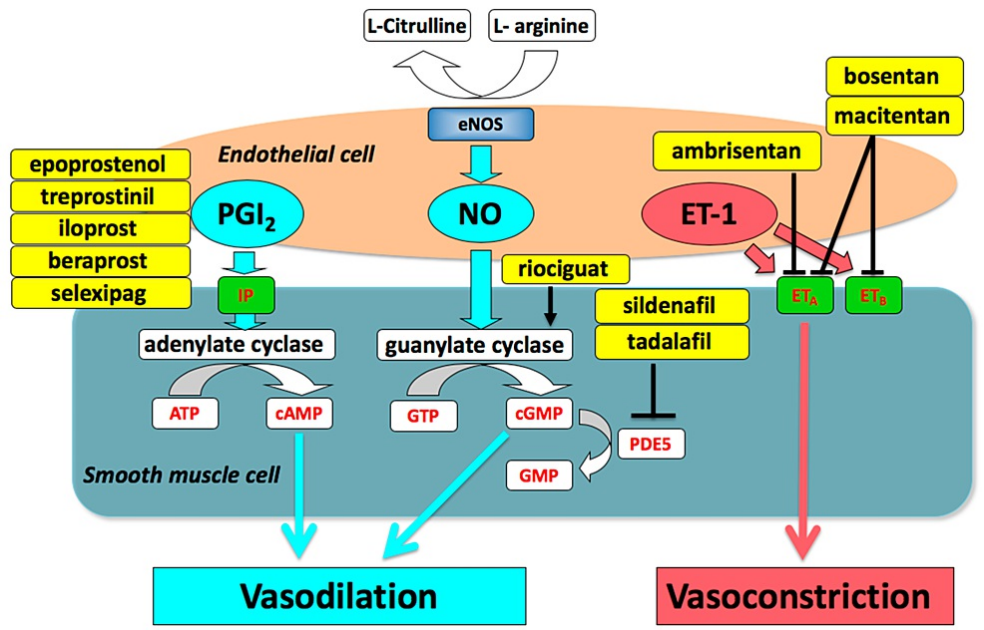
Drugs targeting the three mechanisms involved in pathogenesis of PAH. The image is obtained from Nakamura et al. [21]. eNOS: endothelial nitric oxide synthase; PGI_2_: prostaglandin I_2_; NO: nitric oxide; ET-1: endothelin-1; IP: prostaglandin I2 receptor; ETA: endothelin type A receptor; ETB: endothelin type B receptor; ATP: adenosine triphosphate; cAMP: cyclic adenosine monophosphate; GTP: guanosine triphosphate; cGMP: cyclic guanosine monophosphate; PDE5: phosphodiesterase type 5

The pathway of the vasoactive intestinal peptide (VIP), a neuropeptide of 28 amino acids, is essential for the generation of both water and electrolytes in the stomach and predominantly functions as a neurotransmitter [20]. As a potent systemic and pulmonary vasodilator, VIP also has a slight inhibitory effect on PASMC growth and platelet activation. Mechanisms involved in the pathogenesis of PAH have been well depicted in Figure 2.

Advantages and disadvantages of drugs

Inhalational delivery offers various benefits over conventional medication delivery methods in treating PAH. First, greater pulmonary drug concentrations can be administered by this route, and second, less systemic side effects are observed [22]. Third, gas exchange is improved by inhaled vasodilators. Vasodilator drugs that are administered systemically cause the pulmonary artery vasculature to dilate indiscriminately, resulting in increased blood flow to inadequately ventilated locations and impairing gas exchange. As opposed to this, pulmonary infusion of vasodilators delivered the drugs to the vented loci, wherein its distinct vasodilating action may boost blood supply, enhancing ventilation and perfusion. Lastly, compared with other delivery forms, inhalation can result in better drug absorption, and a quick action start in the lungs, usually within minutes [23]. Due to the relatively lower enzymatic activities of the lungs, inhalation has additional benefits over other methods of pharmacological delivery, including the avoidance of first-pass metabolism [24]. There are drawbacks to the inhaled method as well. Airway symptoms like cough may occur from intolerance to the administration of inhaled medications because of the direct irritating effects of the drugs on the airways. Patients can also not appropriately regulate drug dosage via variable breathing techniques and the challenge of determining the specific quantity of medication that reaches the pulmonary artery of the lung. Due to the difficulties in operating the equipment, pulmonary delivery methods increase the possibility of human error and improper dosage [25]. Most insoluble particles are kept in the mucus of the conducting airways, where they are pushed upward toward the throat by the mucus produced by the metachronous movement of cilia (and ultimately the gastrointestinal system). A factor affecting a drug's capacity to pass this mucous membrane is particle size. Other factors include solubility, lipophilicity, solubility, and charge. Advantages and disadvantages of routes of drug administration are briefly enlisted in Table 2 [26].

**Table 2:** Advantages and disadvantages of various approaches of drug delivery.

Formulations	Advantages	Disadvantages
Nasal spray	They may be formulated in the form of solution and suspension. Exact dose can be delivered via metered dose pumps	Less efficient than nasal drops when human serum albumin is stored in the nostrils
Nasal drops	Simple and convenient to administer	Lack of dose precision
Nasal powders	Absence of preservatives and superior stability	The appropriateness of powder composition depends on the solubility, particle size, aerodynamic properties, and nasal discomfort of active drugs
Nasal gels	High viscosity reduces post-nasal drip, reducing the effect of tastes due to reduced swallowing	Local side effects
Liposomes	Active encapsulation of large and small molecules with high hydrophilicity and pKa values	Production cost is high. Short half-life
Nanoparticles	Deposits are small	Only the smallest nanoparticle penetrates the mucous membrane by paracellular route and in a limited amount

There may be some benefits of using inhaled route of administration in treatment of PAH. People who use nebulizers, metered dose inhalers (MDIs), or dry powdered inhalers (DPIs) to inhale medicine experience less flushing and diarrhea.

Jet Inhalers

When the compartments containing the liquid formulations are triggered, an aerosol mist is created. In these nebulizers, the reservoir is a corrugated tube that, when breathed, produces an aerosol. A large proportion of medicine is lost as a result of aerosol generation. Breath-enhanced jet nebulizers are so-called because they facilitate the patient breathing while inhaling [27].

Inhalers With a Pressurized Metered Dose

Metered-dose inhalers are pressurized devices that employ propellant sprays to deliver medication to the lungs (i.e., MDI) [28]. For individuals with chronic obstructive pulmonary disease and asthma, these are the pharmaceutical administration methods that are most frequently used. Inhalers with dry powder (DPIs) are portable, non-propeller devices as minimal patient cooperation is needed for both breathing and aerosol intake; DPIs address the primary restriction of MDIs.

Future aspects of systematic drug delivery using nanoparticles (nano-DDS)

For the transportation of medications to target organs, nanoparticles (NPs) have been utilized in various innovative delivery methods. Because of their tiny size, NPs can penetrate tissue and be maintained, allowing them to be taken up by the target organ. The composition of NPs can be used to regulate drug release from them [29]. Medication-loaded NPs for local administration can maximize pharmacological effectiveness and reduce adverse drug effects. Every medicine has the potential to be toxic, which might lessen the safe dose and, as a result, the therapeutic efficacy. Using a nanoparticle drug delivery system (nano-DDS) may make medicinal compounds more effective and secure while overcoming limitations such as toxicity, poor water solubility, and limited bioavailability [29]. Although vasodilators, such as prostacyclin (PGI_2_), sGC stimulants, and PDE5 inhibitors have been used to treat PAH effectively [30]. Though IV infusion of prostacyclin or systemically delivery of imatinib have improved quality of life and prolonged survival of patients, but seem to have their side effects and adverse events. Nano-DDS have been suggested as a potential therapeutic option for managing PAH patients. Nano-DDS for lung therapy might increase medication effectiveness and reduce adverse effects.

New Molecular Targets for PAH: A Different Perspective

**Figure 3:**
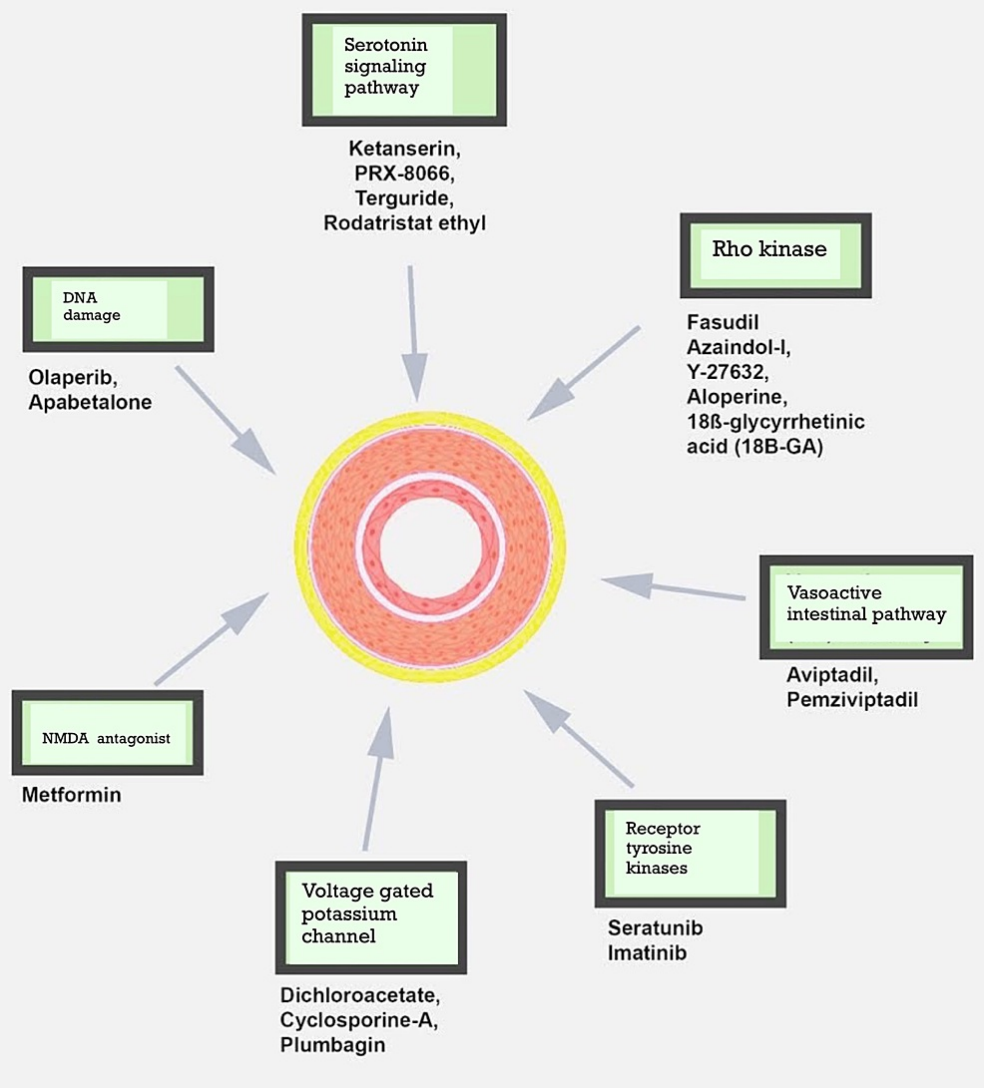
Newer molecular targets for the therapies of pulmonary hypertension. The image is adapted from Dhoble et al. [31]. NMDA: N-methyl-D-aspartate; DNA: deoxyribonucleic acid

Given the limitations of the current PAH therapies, it would be ideal to identify novel molecular pathways that target the illness; and create formulations that efficiently deliver drugs to the target region. Investigating novel molecular targets for the disease is crucial given the various processes underpinning the PAH pathogenesis. More recent molecular pathways may speed up the introduction of newer PAH drugs and eliminate the requirement for the combinational drugs now required to obtain desired treatment effects [31]. Figure 3 shows the various newer molecular targets for the treatment of PAH.

Integrating nanocarriers in PAH

The focus of research is shifting to targeted drug delivery utilizing nanotechnology due to the requirement for the administration of traditional medications at the sick location and the emergence of new targets. It uses the enhanced physiochemical and biological capabilities of substances at the nanoscale, between 1 nm and 1000 nm, simulating the functions of molecules naturally and their interactions with other molecules. In recent years, nano-drug delivery systems have significantly outperformed traditional dosage forms as a medication delivery method for life-threatening and crippling illnesses [32].

Liposomes

In 1995, liposomes were the first nanoscale delivery system employed in a clinical setting to treat diseases, and they have since demonstrated their usefulness in the medical, cosmetic, and culinary industries [33]. Liposomes are lipid bilayer vesicles made of phospholipids that are self-assembled to form an aqueous core. Due to their amphiphilic properties, liposomes may coerce both hydrophilic and hydrophobic medicines in their water cores and lipid layers, respectively. A few advantages of liposome drug-delivery systems include controlled drug release, biocompatibility, biodegradability, biomimetic delivery of drugs, and improved transcriptional activator for gene delivery [33]. Kleemann et al. focused on the aerosol delivery of engineered lipid nanoparticles containing dipalmitoylphosphatidylcholine (DPPC), 1,2-bis diphenylphosphino ethane-polyethylene glycol (DPPE-PEG), and cholesterol for improving the distribution of iloprost in lungs [34]. The polyethylene glycol alteration prolongs circulation time, reducing the need for further doses.

Micelles

These are thermodynamically beneficial supramolecular structures formed when surfactants or polymers self-assemble in an aqueous environment at concentrations over the critical micelle threshold (CMC). The hydrophobic heads travel inward to create the center of the micellar system, and the hydrophilic tails move outward to form the outer shell [35]. This precise pattern of orientation results in size variations between 10 nm and 50 nm. Utilizing polyethylene glycol-tagged stearoyl-phosphoethanolamine micelles with extremely low critical micelles threshold, researchers have examined the effectiveness of numerous drugs, such as paclitaxel and rifampicin for treating a variety of respiratory infections, including cancers and tuberculosis [35].

## Conclusions

Numerous research points to innovative molecular treatments for pulmonary arterial hypertension (PAH) in light of the negative functional alterations linked to the disease and related pathological variability. To create better therapies and drug delivery technologies, it is crucial to investigate the molecular processes underlying the pathophysiology of PAH. However, it is important to identify the relationship between the inhaled formulation and the way the medicine is currently delivered. A conceptual and technological leap will undoubtedly be needed to target medicine delivery in PAH. Drug delivery to a particular PAH site will be extremely effective due to the development of sophisticated inhalation platforms. The diseases of PAH and their negative effects on inhaled medications impact the performance and efficacy of newer formulations. As a result, considerable effort must be made to grasp strict formulation requirements and how they relate to important inhalation targeting novel treatments. Numerous variables might be blamed for these challenges, including the challenge of maximizing therapeutic efficacy and the absence of rigorous assessment of the drug's effect in reversing the underlying pathological vasculature restructuring of lung arterioles. To offer some useful recommendations for the management of PAH, this study's conclusion highlighted the status of pulmonary hypertension and the qualities of inhalation preparations. More pulmonary hypertension therapy options must be created in the future.
